# In Vitro Biocompatibility of Several Children’s Toothpastes on Human Gingival Fibroblasts

**DOI:** 10.3390/ijerph19052954

**Published:** 2022-03-03

**Authors:** María Pilar Pecci-Lloret, Sergio López-García, Francisco Javier Rodríguez-Lozano, Pablo Álvarez-Novoa, David García-Bernal

**Affiliations:** 1 Gerodontology and Special Care Dentistry Unit, Morales Meseguer Hospital, Faculty of Medicine, University of Murcia, 30100 Murcia, Spain; mariapilar.pecci@um.es; 2 Department of Stomatology, Faculty of Medicine and Dentistry, Universitat de Valencia, 46010 Valencia, Spain; sergio.lopez-garcia@uv.es; 3 Cellular Therapy and Hematopoietic Transplant Group, Instituto Murciano de Investigación Biosanitaria Virgen de la Arrixaca, University of Murcia, 30120 Murcia, Spain; david.garcia23@um.es; 4 Department of Surgery and Medical-Surgical Specialties, School of Medicine and Dentistry, Universidade de Santiago de Compostela, 15705 La Coruña, Spain; pablo.alvarez@rai.usc.es; 5 Biochemistry, Molecular Biology and Immunology Department, Faculty of Medicine, University of Murcia, 30100 Murcia, Spain

**Keywords:** children’s toothpastes, biocompatibility, SLS, fluoride

## Abstract

The European Academy of Paediatric Dentistry has recommended fluoride toothpastes from the eruption of the first teeth in children. Toothpastes stay in the mouth in contact with human gingival fibroblasts (hGFs) for a long time. Thus, the objective of this study was to compare the cytotoxicity of five different commonly used children’s toothpastes: Oral B Kids +3 (Procter & Gamble, Alicante, Spain), Fluor Kin Calcium (Kin, Madrid, Spain), PHB Junior (PHB, Barcelona, Spain), Colgate +3 (Colgate Palmolive, Madrid, Spain) and Vitis Kids (Dentaid, Valencia, Spain) on hGFs. The children’s toothpastes were exposed to hGFs at different concentrations (1:1, 1:2, 1:4). Afterwards, several tests were performed: MTT assays, cell cycle analyses, cell cytoskeleton staining assays, apoptosis/necrosis assays, and ICP-MS and ion chromatography. Oral B displayed the lowest cytotoxicity and was the toothpaste with the highest fluoride ion release; meanwhile, the other toothpastes were cytotoxic (*** *p* < 0.0001); Fluor Kin being the one with the lowest fluoride ion release. Among all the toothpastes analyzed, Oral B exhibited the best results in vitro in terms of biocompatibility. Future evaluations, both in vitro and in vivo, are required to confirm the biocompatibility of sodium lauryl sarcosinate and sodium lauryl sulfate containing toothpastes.

## 1. Introduction

Caries is a preventable disease, and one of the groups most affected by this disease are children [1,2]. Early childhood caries has a prevalence of 48%, which has not changed in the last thirty years [3].

Caries prevention starts at home; it is known that dietary sugar exposure should be avoided or reduced [4]. In addition, another essential factor that influences caries prevention is the self-application of fluoride in the form of toothpaste [4,5,6]. Some studies showed that the prevalence of caries falls by up to 30% in patients who use fluoride-containing toothpastes, compared to those who brush without toothpastes or with toothpastes without fluoride [4,5].

It is recommended to use toothpaste in children from the first tooth; the concentration and the quantity are the two parameters that change. Therefore, from the first tooth to two years old, it is recommended to use a toothpaste with at least 1000 ppm, twice daily and in an amount the size of a grain of rice; from two to six years, it is recommended to use the same toothpastes, but in a pea-sized amount; finally, over six years, it is recommended to use a toothpaste with at least 1450 ppm [6,7].

Toothpastes stay at the mouth for the 2 min of brushing and they stay for more time because rinsing is not recommended after regular brushing; thus, fluoride remains in the mouth (saliva and mucosa) for more time, and it will perform its remineralizing function for longer [4,6,7]. Some studies evidenced that the level of fluoride in saliva stays for more time than the two minutes of cleaning [8]. Moreover, it can remain 24 h after application, depending on its concentration [9], and in addition, the concentration in the mucosa is higher than in saliva [10]. Thus, toothpastes are in contact with human gingival fibroblasts for a long time compared to other oral materials [11,12,13]. On the basis of these reasons, some studies investigated the cytotoxicity of different toothpastes [14,15,16], finding that the toxicity varies according to the toothpastes’ composition.

In a general, the composition of toothpastes is based on: fluorides, antiplaque agents, anti-malodor agents, anti-calculus agents, erosion prevention agents, excipients, abrasives, other noteworthy active ingredients, viscosity and rheology modifiers, humectants, sweeteners, coloring, preservatives, water, surfactants and other excipients [17]; some of them being responsible for the toxicity.

Accordingly, the present in vitro study aimed to compare the cytotoxicity of five different commonly used children’s toothpastes: Oral B Kids +3 (Procter & Gamble), Fluor Kin Calcium (Kin), PHB Junior (PHB), Colgate +3 (Colgate Palmolive) and Vitis Kids (Dentaid) on human gingival fibroblasts. The null hypothesis is that none of the toothpastes tested are biocompatible with human gingival fibroblasts.

## 2. Materials and Methods

### 2.1. Preparation of Children’s Toothpaste Eluates

The toothpastes tested were five different commercially available children’s toothpastes: Oral B Kids +3, Fluor Kin Calcium, PHB Junior, Colgate +3 and Vitis Kids. The manufacturer’s data, the composition, and the lot number of each of the materials tested are shown in Table 1.

Eluates of these materials were prepared following ISO 10993-5 recommendations. Therefore, 0.2 g of each toothpaste was mixed with 1 mL of DMEM culture medium (Gibco, Thermo Fisher Scientific, Carlsbad, CA, USA), centrifuged at 4200 rpm, and the supernatant was collected and filtered. This conditioned medium was sterilized by exposure to ultraviolet light for two hours and used undiluted (1:1), half-diluted (1:2), and quarter-diluted (1:4) for the subsequent experiments.

### 2.2. Isolation and Culture of Human Gingival Fibroblasts

Human gingival fibroblasts were obtained from impacted tooth extraction (*n* = 10), following the Ethical Committee of the University of Murcia (ID: 2199/2018). All the individuals signed the consent form according to Helsinki Declaration Guidelines and were informed about the research project.

The excised gingival tissues were immediately digested for 2 h at 37 °C with 3 mg/mL collagenase I (Sigma-Aldrich, St. Louis, MO, USA) and 4 mg/mL dispase II (Invitrogen, Waltham, MA, USA). Then, the cells were cultured in basal growth medium (Dulbecco’s modified Eagle’s medium (Invitrogen)), supplemented with 10% fetal bovine serum (Invitrogen), 1% GlutaMAX (Thermo Fisher Scientific) and 1% penicillin/streptomycin (Invitrogen). The cells were passaged until they reached 80% confluency, and the cells from passages 2–4 were used for this study.

### 2.3. MTT Assays

Assessment of the metabolic activity of hGFs treated with the toothpaste eluates was performed using a colorimetric 3-(4,5-dimethylthiazol-2-yl)-2,5-diphenyltetrazolium bromide (MTT) assay, as previously described [12]. Briefly, hGFs were resuspended in complete growth medium (*w*/*o* red phenol) and plated at 1 × 10^4^ cells/well in 96-well plates (control), or in complete growth medium at different dilutions (1:1, 1:2, 1:4) of each children’s toothpaste and cultured for 2 min, 30 min, 6 h, 24 h or 72 h at 37 °C. Then, MTT reagent (Sigma-Aldrich) at a final concentration of 1 mg/mL was added to the wells, incubated for 4 h at 37 °C and solubilized with dimethyl sulfoxide (DMSO) (Sigma-Aldrich). Afterwards, the formazan production, directly proportional to the cell metabolic activity, was measured in a spectrophotometer (ELx800; Bio-Tek Instruments, Winooski, VT, USA) by the analysis of the absorbance at a wavelength of 570 nm. Three separate experiments using hGFs isolated from three different donors were performed, each carried out in quintuplicate for each children’s toothpaste and controls.

### 2.4. Cell Cycle Analysis

Cell cycle analysis, by measuring the DNA content, is a method that most frequently employs flow cytometry to distinguish cells in the different phases of the cell cycle. The hFGs were cultured with the different toothpaste extracts (1:1, 1:2, 1:4) for 24 h. Then, the cells were collected and fixed in 70% ethanol and incubated with 40 μg/mL of propidium iodide (PI) and 200 μg/mL RNase for DNA content analysis. Fluorescence was measured with a FACScan Flow Cytometer (Becton Dickinson, San Jose, CA, USA) and the percentage of hFGs in the G_0_/G_1_, S and G_2_/M phases was analyzed using Cell Quest (Sebring, FL, USA) and Modfit LT programs (Becton Dickinson, San Jose, CA, USA).

### 2.5. Cell Cytoskeleton Staining

Fluorescent phalloidin labeling was used to evaluate the organization of the F-actin fibers, as well as for possible changes in cell morphology. Briefly, 3 × 10^4^ hGFs were added on glass coverslips, allowed to adhere and spread, and cultured in complete growth medium alone (control) or in complete growth medium containing undiluted (1:1), or 1:2 or 1:4 dilutions of the different toothpaste eluates at 37 °C. After 72 h, the glass coverslips were extensively washed with PBS at 37 °C, fixed in PBS containing 4% formaldehyde for 10 min and permeabilized with PBS containing 0.25% Triton X-100 (Sigma-Aldrich) for 5 min. Afterwards, cells were stained with Invitrogen™ AlexaFluor™ 594-labeled phalloidin (Thermo Fisher Scientific) and 4,6-diamidino-2-phenylindole dihydrochloride (DAPI) (Thermo Fisher Scientific) at r/t in for 30 min, to detect cell F-actin filaments and nuclei, respectively. Representative images were acquired using a Leica TCS SP2 confocal microscope (Leica, Wetzlar, Germany). Three different images were captured in random fields for each material and dilution.

### 2.6. Apoptosis/Necrosis Assays

hGF viability after treatment with the different toothpaste eluates was evaluated by annexin-V-FITC and 7-AAD staining (BD Biosciences) following the manufacturer’s instructions. Briefly, 3 × 10^4^ hGFs were cultured in complete growth medium alone (control) or in 1:1, 1:2 or 1:4 toothpaste dilutions at 37 °C for 72 h. The samples were analyzed in an LSR Fortessa X-20 flow cytometer (Becton Dickinson) within 1 h after staining. Finally, the percentages of live (unstained), early apoptotic (annexin-V-FITC positive), late apoptotic and necrotic (annexin-V-FITC and 7-AAD double-positive) cells were determined. Each condition was analyzed in triplicate.

### 2.7. ICP-MS and Ion Chromatography

Briefly, 2 g of toothpastes were mixed with 1 mL of DMEM culture medium and centrifuged at 4200 rpm. Afterwards, the supernatants were collected and filtered, and the proportion of calcium, potassium, magnesium, sodium, phosphorus, and silica released from each toothpaste was determined using ICP-MS (Agilent 7900 ICP-MS, Agilent, Santa Clara, CA, USA).

Fluoride concentration was analyzed using a Dionex ICS-2100 ion chromatograph (IC) with an AS19 column, using potassium hydroxide as the eluent.

### 2.8. Statistical Analysis

Each experimental condition was assayed three times and evaluated in three independent experiments. Data were expressed as the mean ± standard deviation (SD). Data were analyzed by one-way analysis of variance (ANOVA) followed by Tukey’s post-hoc test for multiple comparisons, using GraphPad Prism software version 8.0.2 (Graph-Pad Software, San Diego, CA, USA); *p* < 0.05 was considered to indicate a statistically significant difference.

## 3. Results

### 3.1. MTT

**Figure 1:**
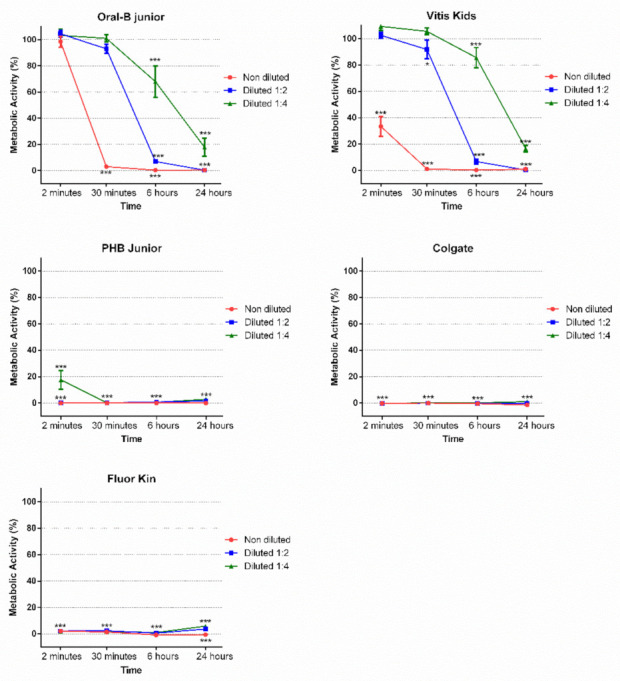
Metabolic activity of hGFs treated with different children’s toothpaste concentrations (1:1, 1:2, 1:4) for 2 min, 30 min, 6 h and 24 h (* *p* < 0.05, *** *p* < 0.001) according to one-way ANOVA and Tukey’s post hoc test.

As shown in Figure 1, the metabolic activity of PHB Junior, Colgate and Fluor Kin were close to 0% in all concentrations, and at all the time points analyzed (2 min, 30 min, 6 h and 24 h), showed significant differences compared to the control group (*** *p* < 0.0001), suggesting a high cytotoxicity. However, Oral B showed an adequate cell metabolic activity (100%) in all concentrations at 2 min. At longer times, undiluted Oral B exhibited null metabolic activity (0%) at 30 min, 6 h and 24 h, with significant differences compared to the control (*** *p* < 0.0001). Furthermore, at 1:2 and 1:4 dilutions of Oral B, the metabolic activity decreased when the dilution was lower and when the time increased. Finally, Vitis Kids showed good results at 1:2 and 1:4, at 2 min and 30 min, but the metabolic activity decreased with time (6 h and 24 h) (*** *p* < 0.0001).

### 3.2. Cell Cycle Analysis

**Figure 2:**
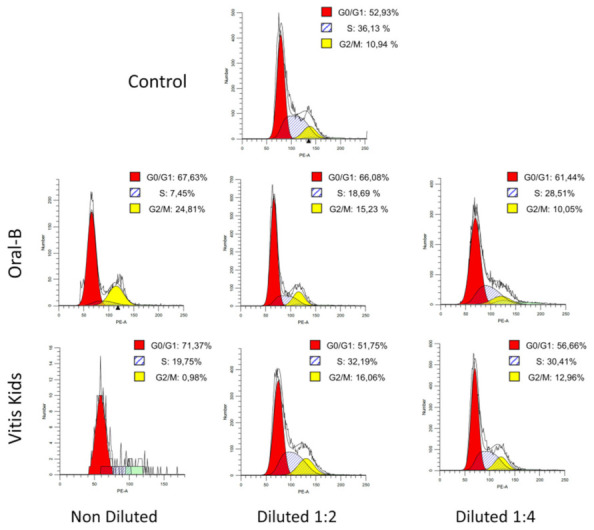
Cell cycle analysis of hGFs after exposure to different children’s toothpaste concentrations (1:1, 1:2, 1:4). Histograms shown are representative of *n* = 3 separate experiments.

Cell cycle phase distributions are presented in Figure 2. Undiluted Oral B and Vitis Kids exhibited the majority of the cells in G_0_/G_1_ phase (67.63% and 71.37%, respectively); Vitis Kids had very few cells in G_2_/M phase (0.98%) and some of them in S phase (19.75%). Meanwhile, Oral B had more cells in G_2_/M phase (24.81%) and less in S phase (7.45%).

On the other hand, 1:2 and 1:4 dilutions of Vitis Kids showed similar cell distribution in each phase, with 51.75% and 56.66% in G_0_/G_1_ phase, 32.19% and 30.41% in S phase and 16.06% and 12.96% in G_2_/M phase, respectively. Oral B showed the majority of the cells in G_0_/G_1_ phase (66.08% and 61.44%) in both concentrations (1:2 and 1:4), followed by S phase but with different concentrations (18.69% and 28.51%) and finally, G_2_/M phase (15.23% and 10.05%).

All the other toothpastes (Fluor Kin, PHB Junior and Colgate) had no results because all of the cells were dead after treatment.

### 3.3. Cell Cytoskeleton Staining

**Figure 3:**
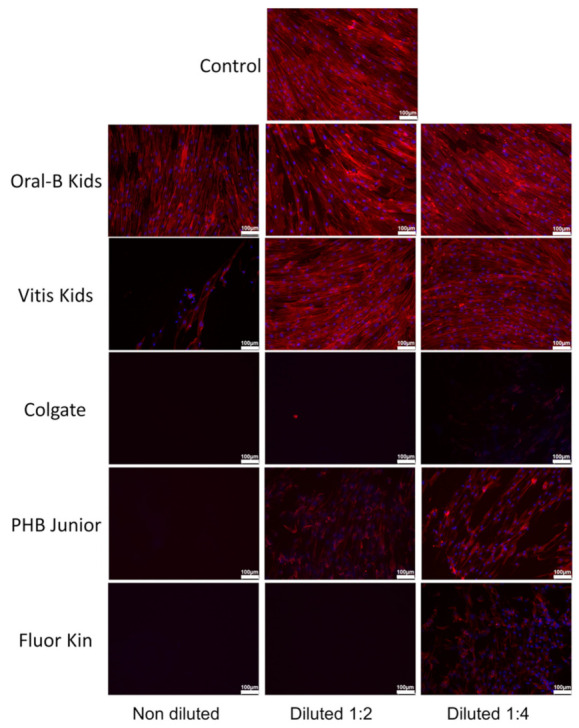
Morphological aspects and cytoskeleton F-actin fibers organization on hGF cultures exposed to different children’s toothpastes by confocal fluorescence microscopy. F-actin fibers were stained with AlexaFluor™ 594-labeled phalloidin (red fluorescence), whereas cell nuclei were counterstained with DAPI (blue fluorescence). Scale bar: 100 μm.

When hGFs were exposed to different toothpastes for 72 h (Figure 3), Oral B cells exhibited a morphology similar to those observed in the control in all the concentrations, with a high quantity and well-attached cells, and an evident actin cytoskeleton and fibroblastic morphology. Vitis Kids only showed cells with a morphology similar to the control and the Oral B groups at 1:2 and 1:4, whereas Colgate did not show any cells at any concentration. Finally, PHB Junior only showed some attached cells at 1:2 and 1:4, whereas Fluor Kin, at 1:4 dilution, displayed a considerably smaller number of attached cells with aberrant morphology.

### 3.4. Apoptosis/Necrosis Assay

**Figure 4:**
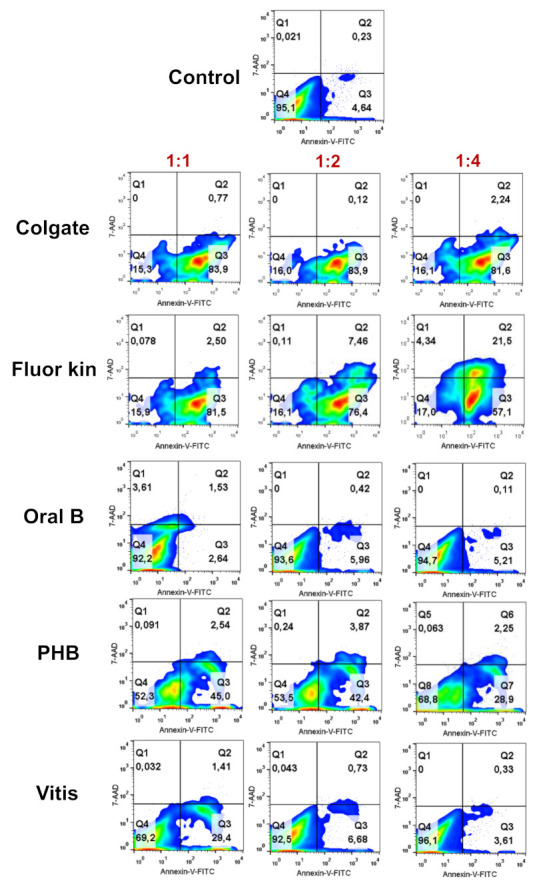
Flow cytometry analysis of cell apoptosis and necrosis induced by the different children’s toothpaste concentrations on hGFs by annexin-V and 7-AAD staining. Numbers inside density plots represent percentages of live (Q4), early apoptotic (Q3), and late apoptotic and necrotic cells (Q1 and Q2) at different concentrations (1:1, 1:2, 1:4). Displayed dot plots are representative of three independent experiments performed in triplicate for each material.

Figure 4 shows the percentage of live and apoptotic/necrotic cells after treatment with different dilutions of children’s toothpastes. Among all of them, Oral B showed a percentage of live cells higher than 92% in all the concentrations, and similar to that observed with the 1:2 and 1:4 concentrations of Vitis. Conversely, Colgate and Fluor Kin only showed 15–17% of live cells in all the concentrations analyzed (1:1, 1:2, 1:4), while PHB displayed an intermediate result, with 52% to 69% of live cells.

### 3.5. ICP-MS and Ion Chromatography

ICP-MS analyzed the ion release as shown in Table 2. The results showed that Ca ion was only detected in Colgate and Fluor Kin, although it is not among the components of Colgate (Table 1). In addition, K ion was present in all the toothpastes, but in a low quantity, with the exception of PHB Junior which displayed 286.6 ppm; only PHB Junior and Fluor Kin had this element in their composition (Table 1). Mg ion was only found in Colgate, Fluor Kin and Vitis Kids, but in a low quantity, and it is not specified in the composition of any of the toothpastes. Na ion was detected in all the toothpastes, with higher levels of Na being found in Oral B (1002.6 ppm). P ion was present in all the toothpastes, with the higher levels detected in Oral B, followed by Fluor Kin. Finally, Si ion was present in all the toothpastes in moderate levels.

The ion chromatography (Table 3) showed the bioavailable ionic fluoride delivered in the different toothpastes. Oral B showed the highest value with 230.42 mg/L of fluoride; meanwhile, Fluor Kin showed the lowest value with 22.91 mg/L of fluoride. The remaining toothpastes displayed a similar ion release. 

## 4. Discussion

In this study, we aimed to assess the cytocompatibility of five toothpastes indicated for use with children. Our findings evidenced different results between the toothpastes analyzed. Four of them were very cytotoxic (Fluor Kin Calcium, PHB Junior, Colgate +3 and Vitis Kids), whereas only Oral B +3 evidenced good biocompatibility results using hGFs as a cellular model.

In terms of cytotoxicity, the composition seems to have an important role. Nowadays, sodium lauryl sulfate (SLS) is the surfactant most used in toothpastes [17]. Cvikl et al. [15] and Birant et al. [16], who analyzed different children’s toothpastes, concluded that all the toothpastes containing sodium lauryl sulfate (SLS) had the worst results, which was in concordance with another study, but using adult’s toothpastes [18]. In addition, Tabatabaei et al. [11] analyzed the ingredients commonly used in toothpastes and agreed that SLS had the worst results. All of these results are in agreement with our findings, being that Oral B is the only toothpaste without SLS in its composition and with the best biocompatibility results. Interestingly, Vita Kids, that contained sodium lauryl sarcosinate instead of sodium lauryl sulfate, exhibited moderate cell viability rates. Therefore, more studies are necessary that analyze the influence of sodium lauryl sarcosinate in the biocompatibility of toothpastes.

Another commonly used surfactant is cocamidopropyl betaine (CAPB) [17]. It has been reported that the presence of CAPB only (without being mixed with another detergent) has been associated with adequate cell viability results [15], consistent with other cytotoxicity studies using adult’s toothpastes [18]. This finding agrees with our results, since Oral B, which had the best results, is the only toothpaste with this surfactant in its composition. Conversely, Tabatabaei et al. [14] reported moderate cytotoxicity with CPAB. Thus, our results could be explained due to the low concentrations of CPAB in these toothpastes.

Sodium benzoate is used as a preservative in toothpastes. Its cytotoxicity was analyzed by Tabatabei et al. [11], who concluded that this component at standard concentration was not toxic for hGFs; among all the components analyzed, it caused the lowest cytotoxicity. These results agree with ours; this preservative is present in Oral B and Vitis Kids, which produced the best results. Paraben is another commonly used preservative which was analyzed that presented low cytotoxicity [14], which is in Oral B. In summary, all the components present in Oral B had the best results, which agrees with our results, being that Oral B is the most biocompatible children’s toothpaste.

The ion chromatography analyzed the bioavailable ionic fluoride delivered in toothpastes. Oral B showed the highest value, being reasonable because it is the toothpaste with the higher concentration of fluoride (1450 ppm compared to 1000 ppm in the other toothpastes), although it releases more than twice the fluoride ions than the others (230.42 mg/L); Levine et al. [19] concluded that the fluoride content is not the same as the bioavailable ionic fluoride, which agrees with our results because Fluor Kin displayed the lowest concentration (22.91 mg/L), around six times lower than Colgate, Vitis Kids and PHB, although it showed the same concentration in ppm compared to these other toothpastes (1000 ppm).

Oral B showed the highest concentration in ppm of fluoride. It also releases the highest amounts of fluoride ions, being the toothpaste with the highest biocompatibility of all the toothpastes analyzed in this study. This means that fluoride does not influence the cytotoxicity of toothpastes.

A limitation of this study is that we have not measured the fluoride ppm of the toothpastes, only the bioavailable ionic fluoride. If the ppm of Fluor Kin was wrong, it could explain why the bioavailable ionic fluoride was less. However, Perez-Silva et al. [20] analyzed the fluoride concentration in eleven toothpastes and the results agree with the manufacturers.

Given these results, it would be interesting to manufacture children’s toothpastes with less cytotoxic surfactants and preservatives, since these are products that are used by growing children. In addition, future research is necessary to assess why the release of ions varies to ensure that all toothpastes release the required fluoride to prevent cavities.

## 5. Conclusions

Among all the toothpastes analyzed, Oral B had the best results in vitro in terms of biocompatibility. Future evaluations, both in vitro and in vivo, are required to confirm the biocompatibility of sodium lauryl sarcosinate and sodium lauryl sulfate containing toothpastes.

## Figures and Tables

**Table 1:** Children’s toothpastes tested.

Material	Manufacturer	Composition	Lot Number
Oral B Kids +3	Procter & Gamble Poligono Industrial El Espartal, 1, 03100, Xixona, Alicante, Spain	Aqua, Hydrated Silica, Cocamidopropyl Betaine, Trisodium Phosphate, Aroma, Cellulose Gum, Sodium Fluoride, Carbomer, Sodium Saccharin, Limonene, Benzyl Alcohol, Sodium Benzoate, Cinnamal, Polysorbate 80, CÑ 42090 (1450 ppm Fluoride)	0213028870
Fluor Kin Calcium	Laboratorios KIN SA Calle Fuerteventura, 4—PLT BJ, San Sebastian de los Reyes, Madrid, Spain	Aqua, Sorbitol, Glycerin, Hydrated Silica, Aroma, Titanium Dioxide, Xanthan Gum, Xylitol, Sodium Monofluorophosphate, Sodium Lauryl Sulfate, Sodium Methylparaben, Calcium Glycerophosphate, Citric Acid, Sodium Propylparaben, Potassium Acesulfame (1000 ppm Fluoride)	20C09
PHB Junior	Laboratorios de Prevención e Higiene Bucal, Castanyer 25, 08022, Barcelona, Spain	Aqua, Sorbitol, Silica, Glycerin, Titanium Dioxide, Sodium Gluconate, Potassium Acesulfame, Sodium Lauryl Sulfate, PEG-40, Hydrogenated Castor Oil, Xanthan Gum, Sodium Fluoride, Sodium Saccharin, Sodium Methylparaben, Tocopheryl Acetate, Lactic Acid, Aroma (1000 ppm Fluoride)	M2005
Colgate 3+	Colgate-Palmolive, C/Quintanavides, 19—Edificio 4. 28050, Madrid, Spain	Sorbitol, Aqua, Hydrated Silica, Xylitol, PEG-12, Cellulose Gum, Benzyl Alcohol, Sodium Lauryl Sulfate, Sodium Fluoride, Aroma (1000 ppm Fluoride)	(L)1007PL112C 1
Vitis Kids	Dentaid Benimaclet, 39, 46120 Alboraia, Valencia, Spain	Aqua, Sorbitol, Silica, Glycerin, Xylitol, Sodium Benzoate, Cellulose Gum, PEG-40, Hydrogenated Castor Oil, Sodium Lauryl Sarcosinate, Propylene Glycol, Sodium Fluoride, Sodium Saccharin, Benzoic Acid, Mica, Titanium Dioxide, Tetrasodium EDTA, Neohesperidin Dihydrochalcone, Aroma, CL16035 (1000 ppm Fluoride)	R2015

**Table 2:** ICP-MS results.

	Ca (318.127 nm) ppm	K (766.491 nm) ppm	Mg (277.983 nm) ppm	Na (588.995 nm) ppm	P (214.914 nm) ppm	Si (251.432 nm) ppm
**Colgate**	1.1	1.1	0.6	459.5	1.4	12.1
**Fluor Kin**	13.8	11	0.8	576.2	270.2	5.9
**PHB Junior**	0	286.6	0	640.1	5.4	20.6
**Oral B Kids**	0	2.3	0	1002.6	334.8	4.6
**Vitis Kids**	0	1.2	0.1	843.1	2.5	10.7

**Table 3:** Ion fluoride release.

Toothpaste	Fluoride Concentration
Colgate	118.14 ± 2.22 mg/L
Oral B Kids	230.42 ± 5.56 mg/L
Vitis Kids	146.75 ± 5.08 mg/L
PHB Junior	163.54 ± 3.03 mg/L
Fluor Kin	22.91 ± 3.00 mg/L

## Data Availability

The data presented in this study are available on request from the corresponding author.
